# Renal Oncocytoma: A Case Report and Review of the Literature

**DOI:** 10.7759/cureus.114925

**Published:** 2026-08-21

**Authors:** Krishna kumar Sah, Dil Sahi Roka, Amit Kumar Sah, Siddhartha Sonu Shah, Xin Meng

**Affiliations:** 1 Radiology, The Third Affiliated Hospital of Qiqihar Medical University, Qiqihar, CHN; 2 Obstetrics and Gynecology, The Third Affiliated Hospital of Qiqihar Medical University, Qiqihar, CHN; 3 Medical Microbiology, Madhesh Institute of Health Sciences, Janakpur, NPL; 4 Pharmacology, Kathmandu University, Dhulikhel, NPL

**Keywords:** histopathology, mri, nephrectomy, renal cell carcinoma, renal oncocytoma

## Abstract

Renal oncocytomas (ROs) are uncommon, benign epithelial tumors of the kidney. We report a case of an asymptomatic RO incidentally detected in a 34-year-old woman that was difficult to differentiate preoperatively from renal cell carcinoma (RCC). Abdominal ultrasonography revealed a right renal mass measuring 2 cm, while MRI showed a cystic-solid nodule without a central scar in the mid region of the right kidney. The patient underwent partial right nephrectomy via the lumbar region without complications and was discharged on the seventh postoperative day. Although MRI showed no central scar, immunohistochemistry and histopathological examination of the specimen demonstrated the characteristic features of RO without evidence of malignancy. This report discusses a small, asymptomatic RO and its differentiation from RCC using radiological imaging, immunohistochemistry, and histopathological examination to establish a definitive diagnosis and avoid misdiagnosis based solely on radiological features.

## Introduction

Renal oncocytoma (RO) is a rare, benign, slowly growing epithelial tumor of the kidney. RO accounts for approximately 3-7% of solid renal tumors, is typically diagnosed between the sixth and seventh decades of life, and is more common in men. Microscopically, typical RO cells are cuboidal, regular, or polygonal in shape, with abundant granular eosinophilic cytoplasm and centrally located round nuclei, with mitoses absent or rarely observed. The cells are arranged in trabecular, nested, mixed, or solid-sheet patterns, separated by delicate fibrovascular stroma [1]. RO arises from the intercalated cells of the collecting ducts of the distal renal tubules and presents asymptomatically in most cases. ROs are commonly smaller than renal cell carcinomas (RCCs), whereas large RO cases are rare. RO generally presents as a unilateral mass with a central scar and a spoke-wheel pattern, and approximately 5% of cases are bilateral [2,3]. The definitive diagnosis of RO is made by histopathological analysis of tumor cells [4]. In this case report, the patient was incidentally found to have unilateral RO during a routine physical examination with ultrasound.

## Case presentation

A 34-year-old woman presented to the hospital for a routine physical examination and was asymptomatic. She denied abdominal pain, lower back pain, nausea or vomiting, dysuria, hematuria, or trauma. Her past medical history was unremarkable, with no history of hypertension, heart disease, diabetes, cerebrovascular disease, mental illness, hepatitis, tuberculosis, malaria, surgery, major trauma, blood transfusion, or food or drug allergies. Her vaccination history was unknown. She had no history of smoking, alcohol consumption, living in epidemic areas, exposure to radioactive or toxic substances, or prostitution. Her weight had not changed significantly over the previous six months. There was no enlargement of superficial lymph nodes anywhere in the body. She denied a family history of cancer. The patient’s vital signs and physical examination were unremarkable. Laboratory test results were within the normal range and were performed as part of the preoperative evaluation (Table 1).

**Table 1:** Laboratory investigation results RBC: red blood cells; WBC: white blood cells; MCV: mean corpuscular volume; BUN: blood urea nitrogen

Parameter	Results	References	Unit
Hemoglobin	13.8	12-16	g/dL
RBC	4.7	4.0-5.4	×10^6 ^cells/mm^3^
WBC	7.6	4.0-10.8	×10^3 ^cells/mm^3^
Platelets	198	150-400	×10^3^/µL
MCV	91	80-98	fl
BUN	4.0	3.2-6.0	mmol/L
Creatinine	78	44-133	µmol/L
Sodium	139	136-142	mEq/L
Potassium	4.4	3.5-5.0	mEq/L
Uric acid	125	89-357	µmol/L

A color Doppler ultrasound examination revealed a mass in the right kidney measuring about 2.0 cm in diameter. MRI of the right kidney revealed a cystic-solid lesion (Figures 1-8).

**Figure 1:**
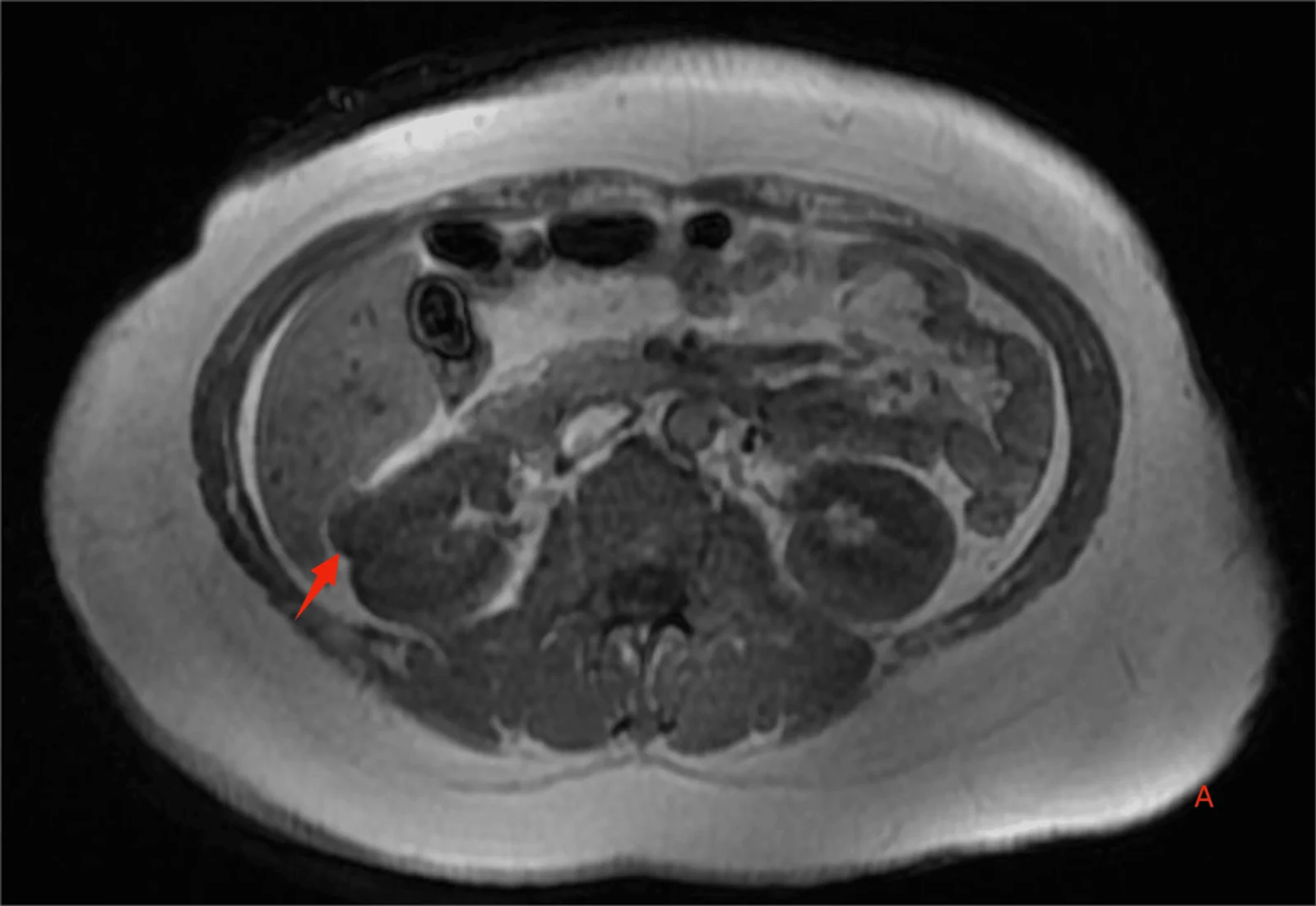
Cystic-solid lesion showing a non-uniform low signal intensity on T1WI (indicated by the arrow) T1WI: T1-weighted imaging

**Figure 2:**
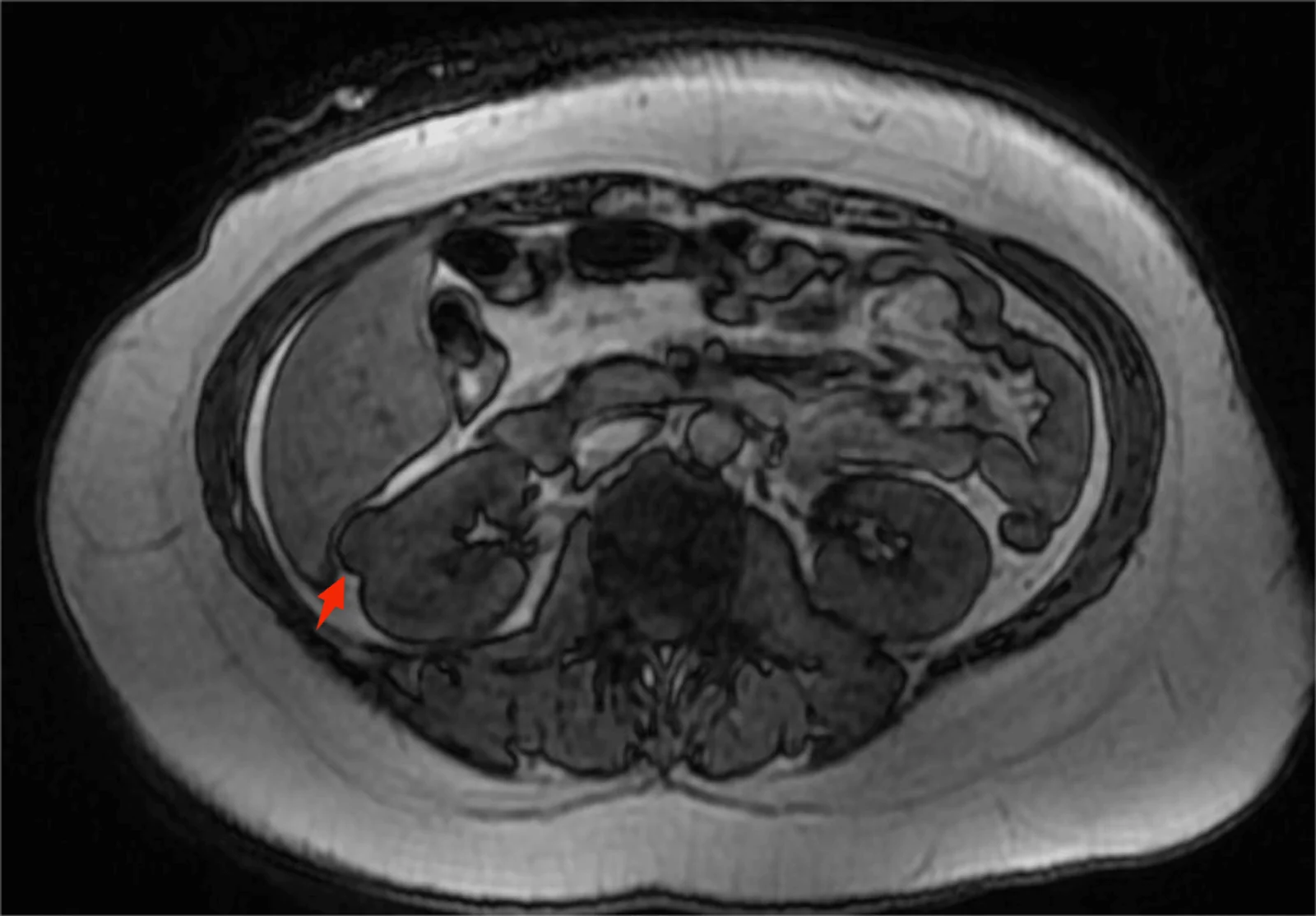
Out-of-phase imaging The arrow indicates that no significant signal reduction was observed

**Figure 3:**
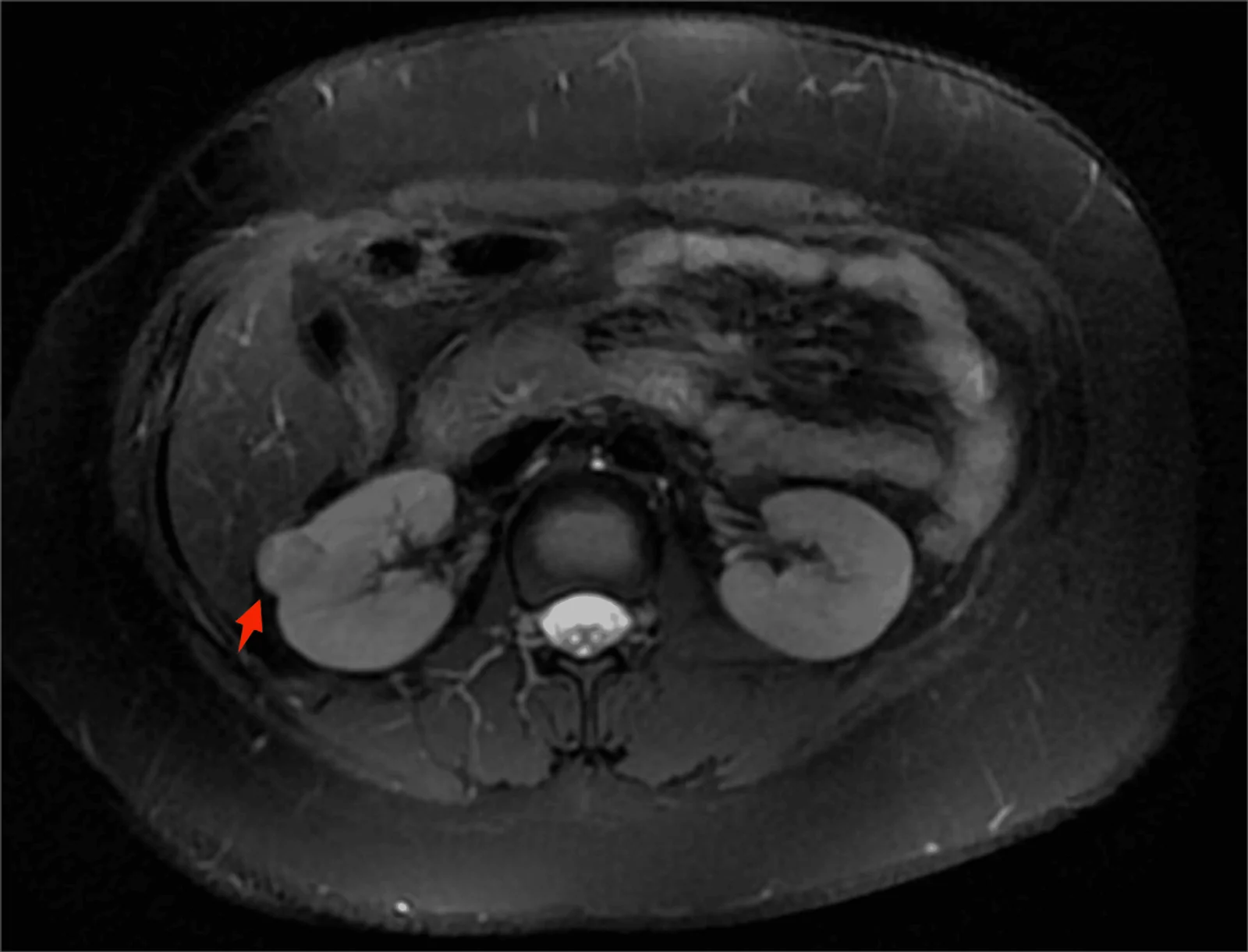
Axial T2WI showing non-uniformity with slightly high signal intensity (arrow) T2WI: T2-weighted imaging

**Figure 4:**
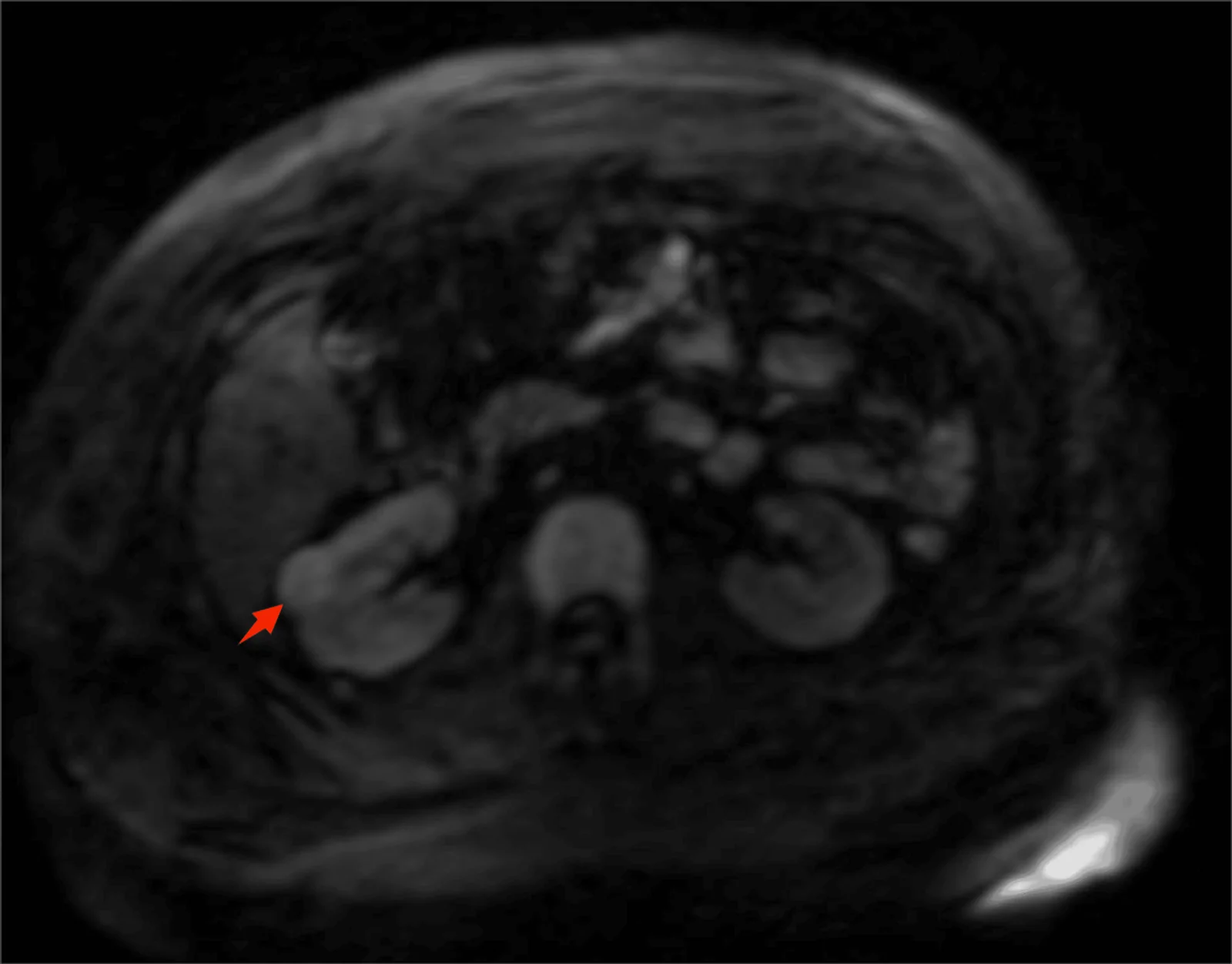
DWI showing high signal intensity (arrow) DWI: diffusion-weighted imaging

**Figure 5:**
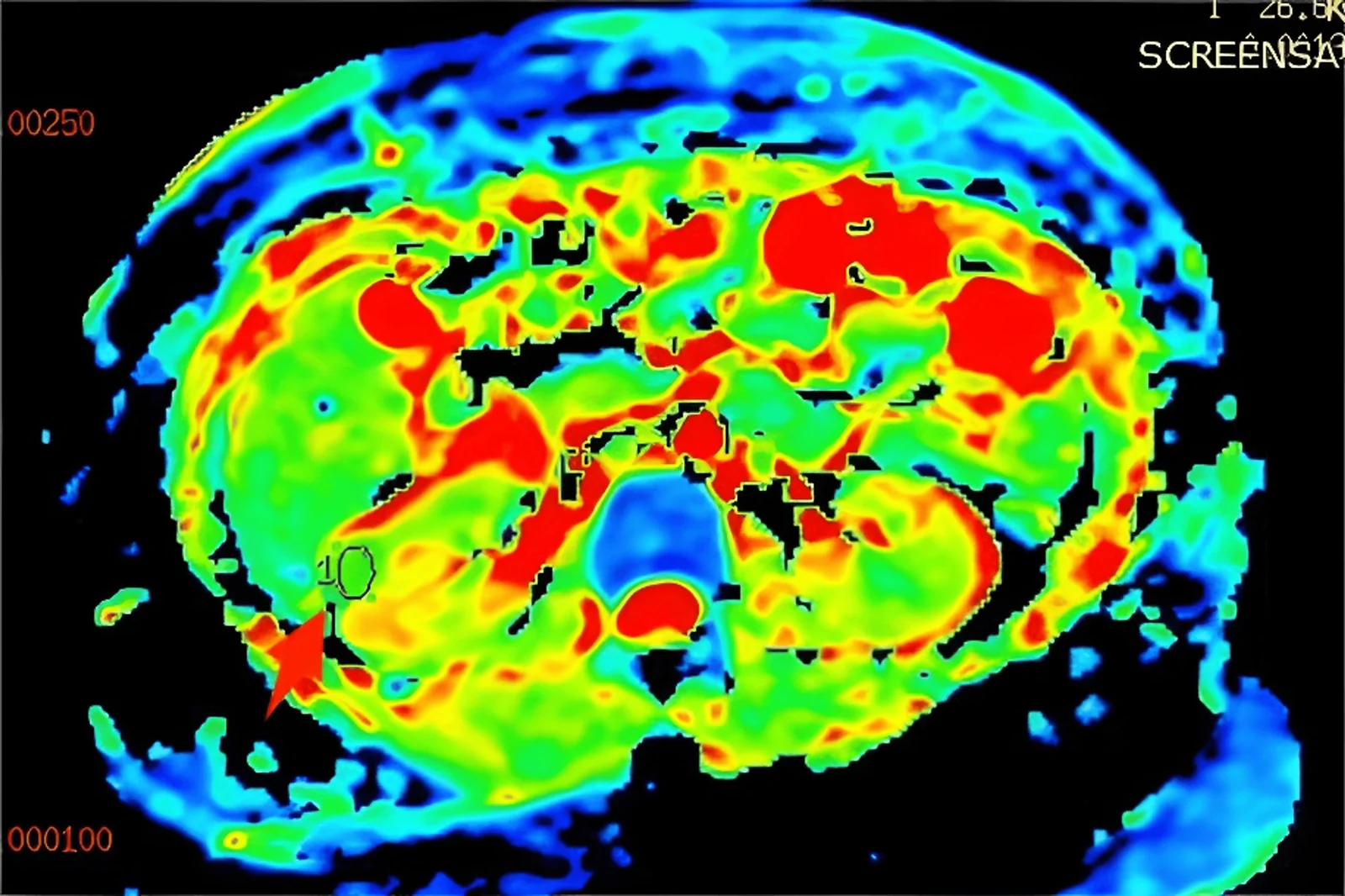
ADC-related findings ADC is reported to be approximately 1.44 × 10⁻³ mm²/s (arrow) ADC: apparent diffusion coefficient

**Figure 6:**
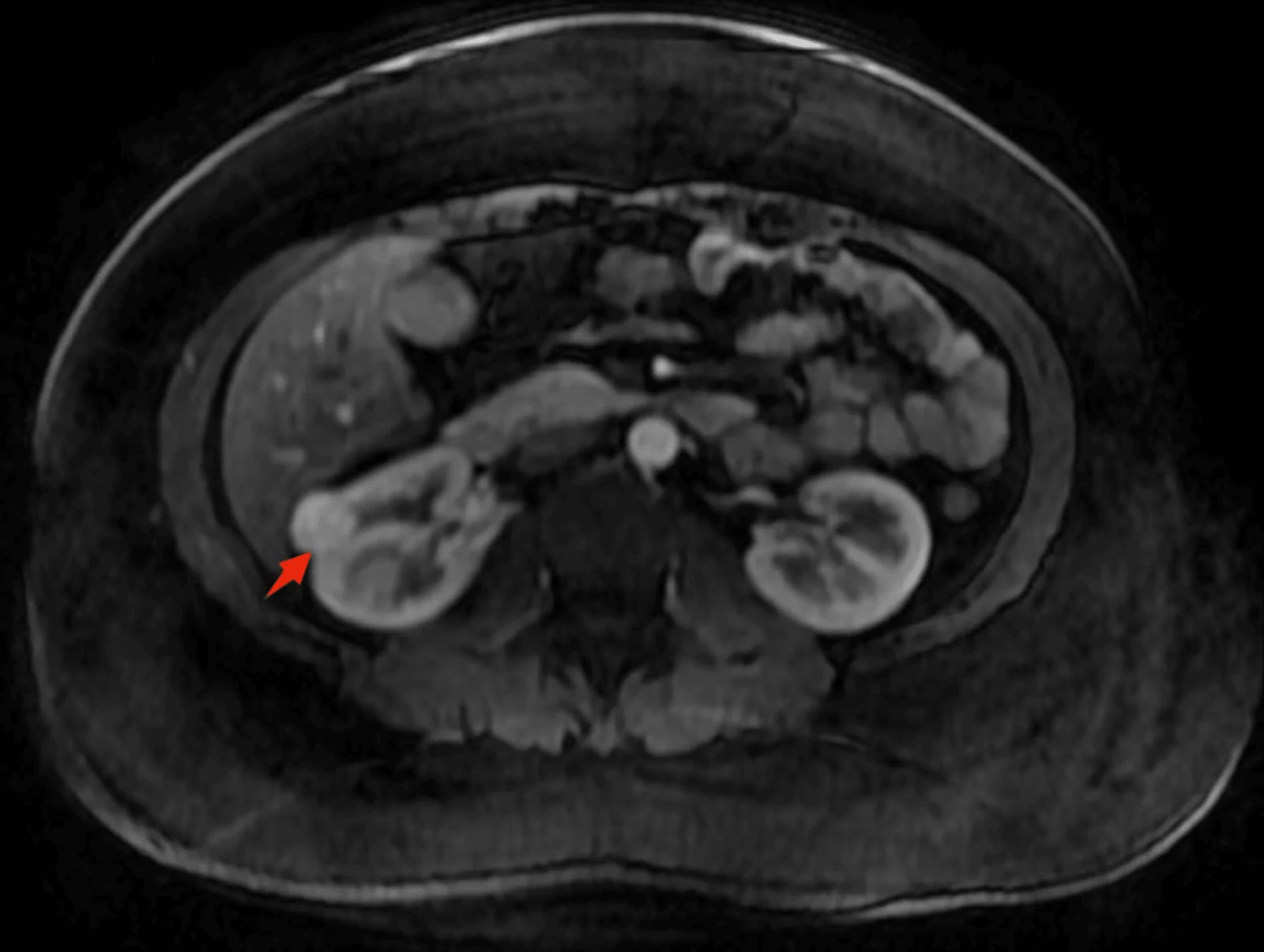
Arterial phase of contrast-enhanced MRI The arrow indicates a lesion with significant and uniform enhancement, suggesting a high blood supply MRI: magnetic resonance imaging

**Figure 7:**
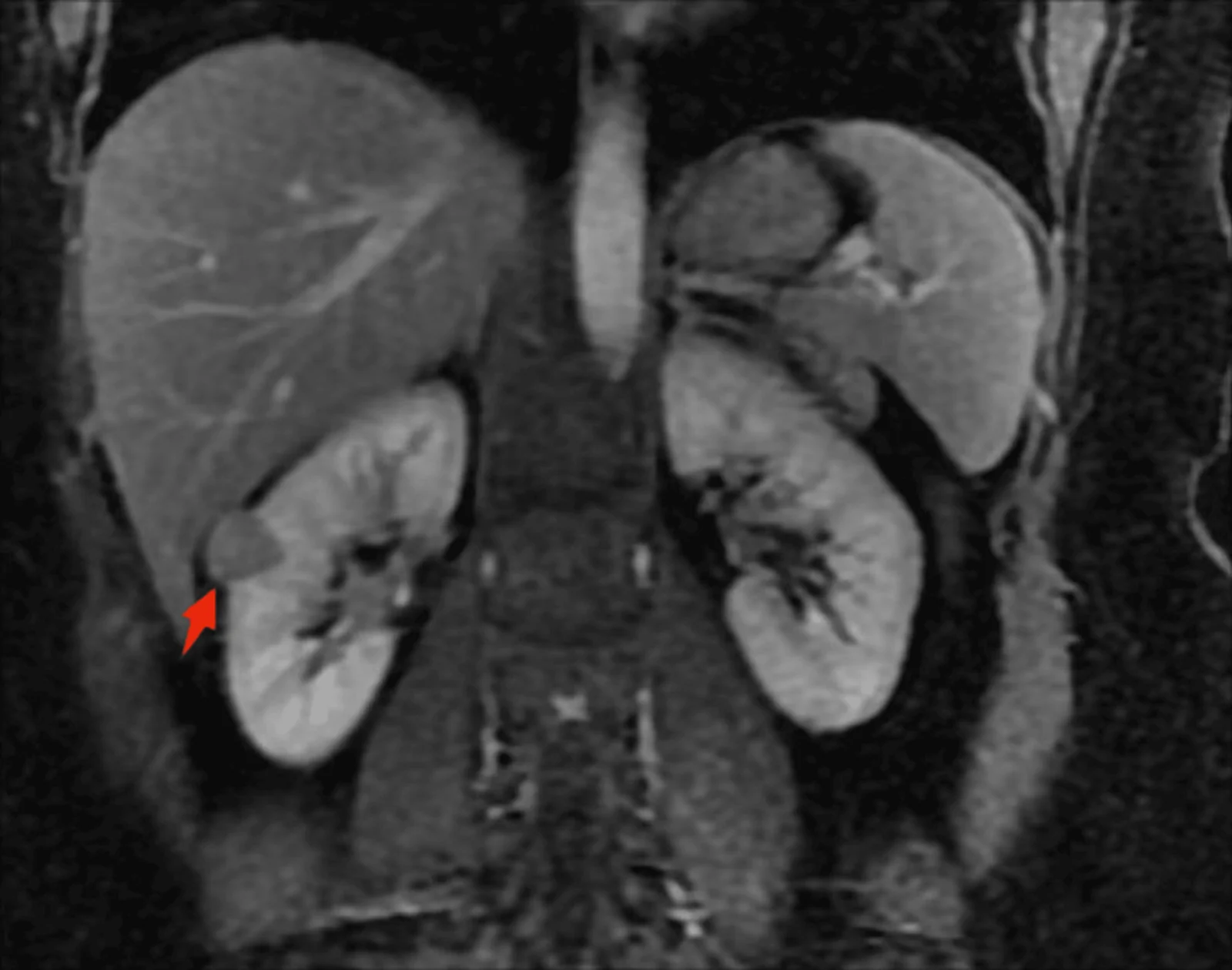
Delayed phase of dynamic contrast-enhanced MRI The mass appears as a relatively low-signal-intensity lesion because the contrast agent is cleared from it, as indicated by the arrow MRI: magnetic resonance imaging

**Figure 8:**
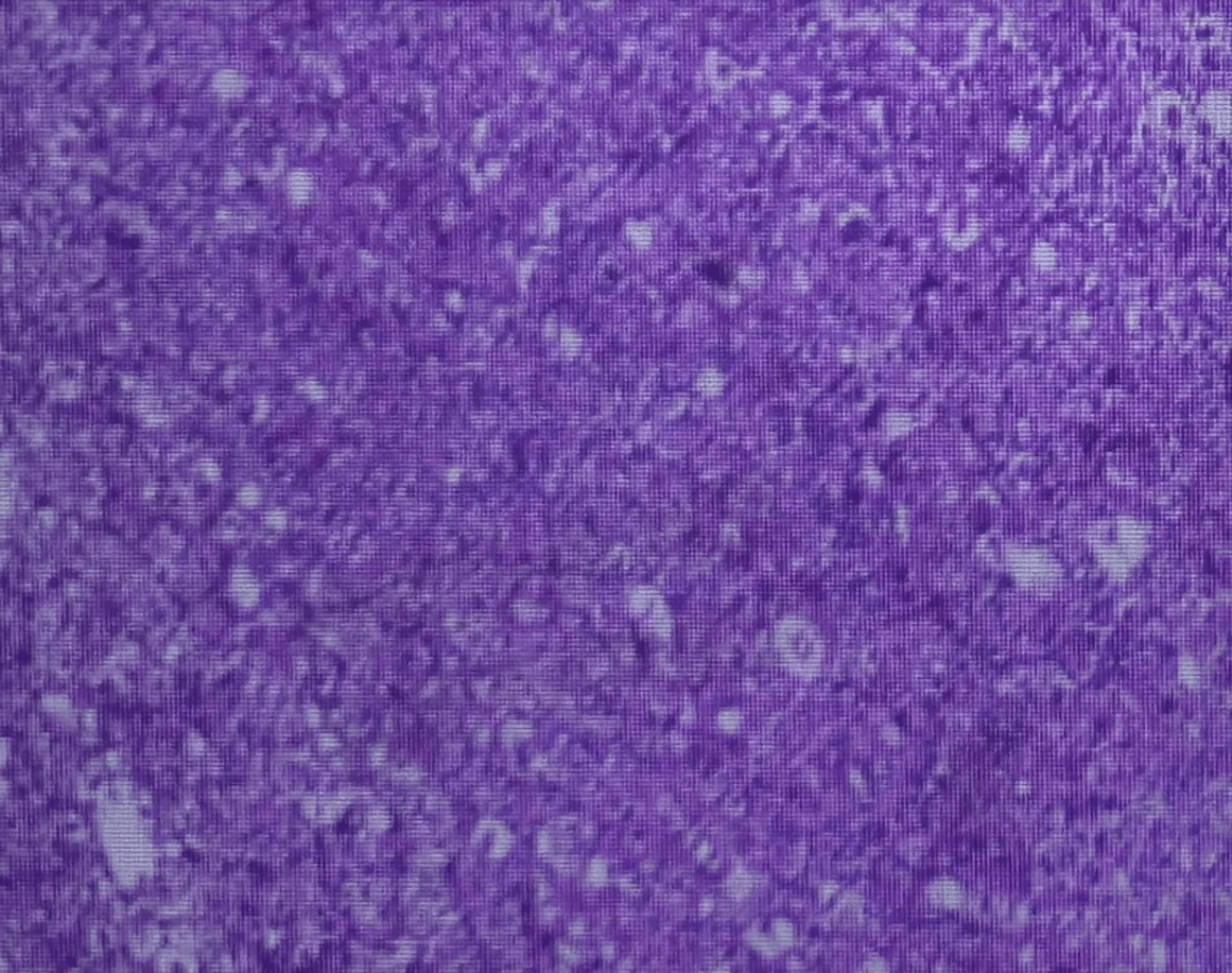
Histopathological slide section with hematoxylin and eosin stain The findings show abundant eosinophilic cytoplasm, and the cells demonstrate round to oval nuclei with basophilic staining. The tumor cells are arranged in sheets/nested architecture

An enhanced MRI examination of the abdomen showed a cystic-solid, partially protruding nodule without a central scar in the midportion of the right kidney, which was suspicious for renal oncocytoma (RO). RO appeared slightly hypointense on axial T1-weighted in-phase and out-of-phase images, with no significant signal drop on out-of-phase images, and slightly hyperintense on T2-weighted images. Diffusion-weighted imaging (DWI) showed hyperintensity with a high apparent diffusion coefficient (ADC) value. RO demonstrated typical hypervascularity, with uniform enhancement in the arterial phase and hypointensity in the delayed phase. The patient successfully underwent robot-assisted laparoscopic partial nephrectomy of the right kidney via the lumbar region under general anesthesia. The right kidney specimen measured 2.5 × 2 × 1.8 cm on gross examination. On the cut surface, a nodule measuring 2 × 2 × 1.8 cm was found, which was grayish-white and of medium consistency. Pathological examination showed an eosinophilic renal tumor surrounded by normal renal tissue. 

Immunohistochemically, the oncocytic cells showed positive staining for PAX8, P504S, CD10, CD117, and SDHB, and negative staining for CA9, vimentin, CK20, TFE3, and CK7. Based on the immunohistochemical, radiological, and histopathological findings, a diagnosis of renal oncocytoma was made. The patient was advised to undergo regular outpatient follow-up. The patient’s clinical presentation, investigation findings, and treatment are summarized in Table 2.

**Table 2:** Timeline summarizing the patient's clinical course MRI: magnetic resonance imaging

Days	Clinical event
Day 1: January 15, 2026	A 34-year-old patient visited the hospital for a routine check-up. Incidentally, on ultrasound, a mass was found in the right kidney
Day 2: January 16, 2026	MRI examination revealed a nodule in the right kidney suggestive of renal cancer after five days
Day 8: January 22, 2026	Patient admitted to the hospital for four days for further treatment
Day 13: January 27, 2026	Partial nephrectomy was done under general anesthesia, and a specimen was collected for histopathological investigation. Then the patient was shifted to the post-surgery ward and stayed for six days. The patient was diagnosed with renal oncocytoma
Day 20: February 3, 2026	The patient was discharged and advised to attend regular outpatient follow-up examinations

## Discussion

Zippel first described RO in 1942, and in 1976, Klein and Valensi reported a series of tumor cases and discussed the benign and pathological features of RO. RO is a benign neoplasm of the kidney. The diagnosis of RO can be aided by radiological imaging modalities, such as ultrasound, CT, and MRI. On axial T1-weighted imaging, RO shows slight hypointensity, whereas on T2-weighted imaging, it shows slight hyperintensity. The central stellate sign and spoke-wheel appearance are characteristic features observed on CT and MRI, although these characteristics are not diagnostic of RO. RO is associated with three genetic abnormalities, although no chromosomal abnormalities have been identified in some cases.

The associated genetic changes related to RO include loss of the Y chromosome or monosomy 1, translocation in the 11q13 region, and congenital abnormalities such as trisomy, monosomy, or loss of heterozygosity. These genetic changes are characteristic of RO and are not observed in RCC [5]. No congenital abnormalities were observed in this case. On gross examination, clear cell RCC appears golden yellow, whereas RO appears tan. In this study, CK7 was positive in 10% of ROs, compared with 73% of chromophobe renal cell carcinomas (ChRCCs). When ChRCC and RO were compared with RCC subtypes, CD117 was positive in both ChRCC and RO but negative in most RCC subtypes. However, the immunohistochemical and histopathological findings of the oncocytic cells confirmed the diagnosis of RO rather than RCC [6].

ChRCC, especially the eosinophilic variant, is an important differential diagnosis for RO because both tumors share gross and microscopic features, including a brown appearance and a central scar. However, ChRCC often exhibits features such as distinct cell borders, perinuclear halos, irregular nuclei, and a higher frequency of binucleated and multinucleated cells. Histologically, RO cells are uniformly arranged, with round nuclei, a compact nested growth pattern, and an absence of perinuclear halos. In addition, immunohistochemistry (IHC) typically shows positivity for CD117 and CK7 in ChRCC, whereas RO is positive for CD117 and negative for CK7. Thus, IHC and histopathological analyses can differentiate ChRCC from RO and confirm the diagnosis of RO [7].

Papillary adenomas are benign renal tubular epithelial neoplasms commonly located in the renal cortex. Their diameter is less than 15 mm, and they lack a pseudocapsule; they are considered potential precursors of papillary RCC. In contrast, RO typically appears as a well-circumscribed tumor with a mahogany-tan color and firm consistency, with a central stellate scar present in approximately one-third of cases. Histologically, the tumor is composed of oncocytic cells arranged in solid nests, with round nuclei. On IHC, oncocytic cells show positive staining for CD117 and negative staining for CK7 and CD10. These features can mimic variants of RCC, making differentiation difficult; therefore, such masses may be treated with radical or partial nephrectomy [8]. Although RO is rare, Omiyale et al. reported 159 resected cases of RO, among which seven showed vascular invasion, 10 showed perinephric fat invasion, and three showed both vascular and perinephric invasion. These findings were identified as atypical features of RO and may suggest malignant potential similar to that seen in RCC [9].

Some patients with oncocytoma present with typical symptoms, such as flank pain, hematuria, and a palpable mass. In addition, some cases are entirely asymptomatic and are diagnosed incidentally during imaging examinations. The standard treatment of choice for RO is nephrectomy. RO measuring less than 4 cm is generally treated with partial nephrectomy, whereas larger tumors are treated with radical nephrectomy [10,11]. In this case, the patient with RO was asymptomatic and underwent partial nephrectomy. Birt-Hogg-Dubé (BHD) syndrome is associated with hybrid oncocytic/chromophobe tumors (HOCTs). Histologically, HOCTs mimic the features of RO and ChRCC. IHC of Birt-Hogg-Dubé-associated tumors shows positive staining for CK7. Compared with these tumors, RO shows negative staining for CK7, supporting the diagnosis of RO [12]. Lastly, this report highlights the differences between RO and other RCCs, as shown in Table 3 [7,8,13-15].

**Table 3:** Histopathological and immunochemical differences between RO and RCC variants RO: renal oncocytoma; RCC: renal cell carcinoma; ChRCC: chromophobe renal cell carcinoma; papillary RCC: papillary renal cell carcinoma; cc-RCC: clear cell renal cell carcinoma

Features	RO	ChRCC	Papillary RCC	Cc-RCC
Microscopic	Mahogany-tan colored mass, with present or absent central scar	Brown appearance, usually with a sharply defined cell border of the mass	Well-demarcated partly cystic/solid yellowish soft mass with necrotic and hemorrhagic areas	With typical golden yellow
Architecture	Nested, tubulocystic	Solid, nested, trabecular	Tubopapillary or papillary pattern	Nested, tubular, surrounded by sinusoidal vessels
Cytoplasm	Eosinophilic	Eosinophilic	Scant/pale eosinophilic	Clear cytoplasm eosinophilic
Nuclei	Round nuclei, lack of perinuclear halos	Irregular, perinuclear halos	Round nuclei with pseudostratification	Nuclei vary from low to high grade
Ck7	Negative	Positive	Positive	Negative
CD10	Positive/negative	Negative	Positive	Positive
CD117	Positive	Positive	Negative	Negative
Vimentin	Positive/negative	Negative	Positive	Positive

A literature review of RO cases according to age, sex, tumor size, location, presenting complaints, and treatment over five years is presented in Table 4.

**Table 4:** Renal oncocytoma cases in the literature

Study	Age, years	Sex	Tumor size, cm	Location	Complaint	Treatment	Year
Bahadori et al. [8]	63	Male	11 × 11 × 9	Right kidney	Flank pain	Open right radical nephrectomy	2021
Sathe et al. [16]	66	Male	3.0, 3.3	Right kidney, left kidney	Flank/lower back pain	Bilateral partial nephrectomy	2022
Kalariya [17]	37	Male	19.9 × 14.2	Left kidney	Abdominal mass	Open left radical nephrectomy	2023
Webster et al. [3]	29	Female	12.3	Right kidney	Abdominal mass	Open right radical nephrectomy	2024
Ou et al. [18]	52	Female	3.8 × 3.5	Left kidney	Asymptomatic	Laparoscopic partial left nephrectomy	2025
Kalfoutzou et al. [12]	82	Female	5.5 × 3.4	Left kidney	Hematuria	Cryoablation	2026
Current case	34	Female	2.0	Right kidney	Asymptomatic	Laparoscopic partial right nephrectomy	2026

## Conclusions

This case report concludes that although RO is benign, asymptomatic, small in size, and may not exhibit all the typical radiological features, a definitive diagnosis requires IHC and histopathological examination. Radiological examination alone is insufficient to differentiate RO from RCC. The main discussion focuses on the imaging features of renal eosinophilic tumors. The findings indicate that the imaging features of renal eosinophilic tumors can mimic those of RCC, making preoperative diagnosis challenging. Therefore, a definitive diagnosis of RO should be established by integrating genetic, IHC, histopathological, and radiological findings. These findings can facilitate accurate diagnosis and appropriate treatment, prevent unnecessary extensive interventions, and reduce the risk of misdiagnosis as RCC, thereby contributing to a favorable prognosis and improved patient outcomes.
